# Longitudinal randomised controlled trials in rehabilitation post-stroke: a systematic review on the quality of reporting and use of baseline outcome values

**DOI:** 10.1186/s12883-015-0344-y

**Published:** 2015-07-01

**Authors:** Odile Sauzet, Maren Kleine, Anke Menzel-Begemann, Anne-Kathrin Exner

**Affiliations:** AG Epidemiology & International Public Health, School of Public Health, Bielefeld University, PO. Box 10 01 31, 33501 Bielefeld, Germany; Faculty of Nursing and Health, University of Applied Science, Münster, Leonardo Campus 8, 48149 Münster, Germany

**Keywords:** Stroke, Rehabilitation, Physical functioning, Longitudinal analysis, Baseline values, Regression

## Abstract

**Background:**

The World Health Organisation stresses the need to collect high quality longitudinal data on rehabilitation and to improve the comparability between studies. This implies using all the information available and transparent reporting. We therefore investigated the quality of reported or planned randomised controlled trials on rehabilitation post-stroke with a repeated measure of physical functioning, provided recommendations on the presentation of results using regression parameters, and focused on the difficulties of adjustment for baseline outcome measures.

**Methods:**

We performed a systematic review of the literature from 2011 to 2013 and collected information on the way data was analysed. Moreover we described various approaches to analyse the data using mixed models illustrated with real data.

**Results:**

Eighty-four eligible studies were identified of which 61 % (51/84) failed to analyse the data longitudinally. Moreover, for 30 % (25/83) the method for adjustment for baseline is not known or not existent. Using real data we were able to show how much difference in results an adjustment for baseline data can make. We showed how to provide interpretable intervention effects using regression coefficients while making use of all the information available in the data.

**Conclusions:**

Our review showed that improvements were needed in the analysis of longitudinal trials in rehabilitation post-stroke in order to maximise the use of collected data and improve comparability between studies. Reporting fully the method used (including baseline adjustment) and using methods like mixed models could easily achieve this.

**Electronic supplementary material:**

The online version of this article (doi:10.1186/s12883-015-0344-y) contains supplementary material, which is available to authorized users.

## Background

In 2011, the World Health Organisation (WHO) published their World Report on Disability [1], providing a framework “for disability data collection related to policy goals of participation, inclusion, and health. [Using it] will help create better data design and also ensure that different sources of data relate well to each other” (p. 45). In the rehabilitation chapter of this report, the lack of randomised trials in rehabilitation research is mentioned and the necessity of collecting comparable outcomes from various sources is pointed out. The report mentions the importance of longitudinal data to understand the “dynamic of disability”. Consequently, it is important in rehabilitation research not only to collect quality data but also to make the best use of it. This includes using all the (statistical) information contained in the data collected, providing the maximal transparency in the description of the methodology, and presenting informative intervention effects.

In order to reflect the dynamic nature of an intervention, the analysis of repeated measures must take the longitudinal nature of the data into account. This presents some difficulties due to the dependence of the measures reported by the same patients. Another less well known difficulty concerns adjusting the effect of intervention for the reduction to mean using baseline outcome values [2]. Moreover, the interpretability of results is paramount for the comparability between studies. Reporting regression parameters with confidence intervals rather than p-values allows the interpretation of the effectiveness of an intervention in term of outcome measures. But this form of reporting, however, is done rarely [3, 4].

The aim of this paper is to present the results of a systematic review of the analysis of measures of physical functioning in randomised controlled trials evaluating interventions in rehabilitation post-stroke. The reasons some approaches are sub-optimal are discussed and we provide recommendations on how to present results using regression coefficients and confidence intervals [5–7]. Those recommendations are illustrated with data from the BOMeN study (Berufliche Orientierung in der Medizinischen Neurorehabilitation [Occupational Orientation in Medical Neurorehabilitation]), a RCT to evaluate the effectiveness of a return to work oriented intervention during residential rehabilitation of stroke and brain damaged patients [8, 9].

## Methods

### Review

In December 2013, the databases Medline, Medpilot, Cochrane Library, and Scopus/SciVerse were searched for articles reporting RCTs or protocols of RCTs on the rehabilitation of stroke patients with a measure of physical functioning. Studies with only one post-intervention measure, no measure of physical functioning, and brain injuries not due to a stroke were excluded from the review. Systematic reviews were also excluded. In order to reflect recent practices, we restricted our search to articles published in 2011 or later. The MeSH terms are given in the online supplement, please see Additional file 1. All extracted studies were screened independently by two of the authors for eligibility by reading the title and abstract. The full texts of all eligible studies were obtained.

Data were collected using a form piloted for consistency, independently by two of the authors and when entries were in disagreement, the articles were checked further. The full list of items extracted from the studies can be seen in Tables 1, 2 and 3. It included background information on the study among which if a baseline measure of physical functioning was collected, whether the data collected were analysed longitudinally, and the method of statistical analysis. It was recorded whether a method of adjustment for baseline measures was described and if an intervention effect was reported.

**Table 1:** Description of studies

Body function outcome	Primary only	19 % (16/84)
	Secondary only	6 % (5/84)
	Primary and secondary	56 % (47/84)
	Unclear	13 % (11/84)
Multiple primary outcomes		29 % (24/84)
Number of arms	2	81 % (68/84)
Number of patients per arm	Median (range)	18.8 (6–182)
Type of study	Comparison of treatment	35 % (29/84)
	Comparison with placebo/usual care	80 % (67/84)
	Cross-over design	5 % (4/84)
Duration of follow-up	median (range) of duration	3 (0.3–60) months
Number of follow-up measures	2	55 % (46/84)
	4	35 % (29/84)
	Other (3,5, unclear)	11 % (9/84)

**Table 2:** Method of analysis

Measurement of outcome	Baseline- 1. After intervention- 2. Follow-up	46 % (39/84)
	Other^a^	54 % (45/84)
^a^More follow-ups after intervention or assessment during intervention		
Repeated measure data was analysed	Cross sectional at each time-point	38 % (32/84)
	Longitudinal	39 % (33/84)
	Both longitudinal and cross sectional	8 % (7/84)
	Repeated data not fully analysed	14 % (12/84)
Method of analysis		
Cross sectional	*t*-test	22 % (10/46)
	ANOVA/ANCOVA	35 % (16/46)
	Regression	7 % (3/46)
	Non parametric test/dichotomised data	37 % (17/46)
Correction for multiple testing due to repeated measures		76 % (31/41)
Longitudinal	Mixed Model	21 % (7/33)
	Repeated measure ANOVA	72 % (24/33)
	Generalised Estimating Equations	6 % (2/33)
Results presented		
	Mean (SD)^b^ at each time-point per group	66 % (47/71)
	F values (for ANOVA/ANCOVA)	54 % (19/35)
	Regression coefficients	71 % (6/12)
	None	8 % (6/71^a^)

^a^13 of the 84 studies were protocols. ^b^Standard deviation

**Table 3:** Use of baseline data in the primary analyse of physical functioning

Baseline data	Collected	99 % (83/84)
Method of adjustment	Mentioned in Methods	64 % (53/83)
	If not, mentioned in Results	6 % (5/83)
	No adjustment	13 % (11/83)
	Unknown	17 % (14/83)
Use of baseline data in the	Difference from baseline	33 % (19/58)
analysis	Covariate	31 % (18/58)
	Time-point	29 % (17/58)
	Used to compute a dichotomised outcome	3 % (2/58)
	Unclear	3 % (2/58)

The results of the review are presented in descriptive tables with absolute and relative numbers of articles for each item. The report of this review follows the PRISMA checklist [10]. This article being based on a review of the literature and is methodological in nature therefore no ethical approval was required.

### Models for the analysis of longitudinal data on rehabilitation

The discussion of the systematic review’s results is illustrated with examples and recommendations using mixed models. We show that the intervention effects can be reported using regression parameters. We provide suggestions on the presentation of method and results illustrated with data from the BOMeN study, analysed using Stata 12 [11]. The BoMeN study (Berufliche Orientierung in der Medizinischen Neurorehabilitation [Occupational Orientation in Medical Neurorehabilitation]), was a RCT performed from 2007 to 2009 in two residential neurological rehabilitation clinics in Germany which evaluated the effectiveness of a return to work oriented intervention during residential rehabilitation of stroke and brain damaged patients. For the BoMeN study, the approvals of the ethic committee of the Medical chamber of Westfalen-Lippe and of the Faculty of Medicine of the Westfälischen Wilhelms-Universität Münster were obtained. Patients recruited included 93 women and 205 men aged 22 to 60 years and 15 to 60 years respectively. The total duration of follow-up was 15 months after the rehabilitation was concluded. The intervention consisted among other in a patient education programme and a better inclusion of workplace related needs in the therapeutic plan. For more detail see [8, 9]. While the primary aim of the study was to compare proportions of patients in work at each time point, the questionnaire FS-36 was also used to collect information about quality of life. The questionnaire has been answered at at least one follow-up time by 295 patients. We computed the physical functioning sub-score and used in the examples presented here.

## Results

We identified 84 eligible studies, 13 of which were protocols. The complete flowchart is available given as online supplement, please see Additional file 2. The study characteristics are presented in Table 1. Most studies had a measure of physical functioning as a primary outcome (68/84, 81 %) and 29 % (24/84) presented multiple primary outcomes in line with the recommendation of the WHO report on disability to reflect the diversity of the aspect of the International Classification of Functioning.

All results regarding the statistical analysis are presented in Table 2. Only 39 % (33/84) of studies performed a longitudinal analysis of the data. Other studies analysed the data cross-sectionally (32/84, 38 %), mostly at each measure time-point, thus losing the dynamics contained in the data. In twelve studies (14 %) not all the collected longitudinal data was analysed, thus a considerable amount of information available was ignored.

The results presented for the 71 studies which were not protocols included mostly mean and standard deviations at each time-point and for each group (47/71, 66 %) but no overall effect of the intervention over time was ever presented. For almost a third of studies (25/83, 30 %) it is unclear if baseline outcome values were used in the analysis. For data analysed longitudinally, the most common model estimated a time-group interaction and 14 studies from 21 used baseline as a time-point in the regression. Two studies used a change from baseline in a longitudinal analysis which means that outcomes at different time-points were not comparable. In all others, longitudinal analysis baseline was covariate in the model.

## Discussion

Our review has shown that baseline measures are consistently collected but not always adjusted for. Moreover, 52 % of studies ignored the longitudinal nature of the data among which 14 % did not use all the follow-up data available. This is evidence that a lot of the information collected and available is not used. Moreover, analysis based on the analysis of variance (for example repeated measures ANOVA) seems to remain popular even when the limitation of these relative to regression based method like mixed models have been often presented in the literature [4, 12].

We outline the difference between analysis of variance (ANOVA) and regression models. The ANOVA is a generalisation of the *t*-test and compare the means of several groups of patients. A regression model provides a relationship between an outcome and some predictors with an error term. The regression coefficient for the group effect is the effect of the intervention.

Mixed models are regression models in which the non-independence of the measures taken on the same patient is accounted for, in its simplest form, by allowing the constant in the model (intercept) to vary between patients (random intercept model). This is described as a random effect. In such models the effect of the intervention is the same for all and is given by the coefficient obtained for the intervention group. This is a fixed effect. Because the model consists of fixed and random effect, they are called mixed models. In studies with long-term community based follow-up, the number of patient with some non-completed follow-up can be large. Valuable information is nevertheless available for those patients. Repeated measures ANOVA can only take into account patients with all follow-up measurements [4]. Mixed models use data from all patients with at least one post baseline measure [5–7], thus making the most of the data available. Of course, missing measures and loss-to-follow-up should be avoided in the first place by careful planning and by developing effort to track down patients who have moved. Another advantage of mixed model is that a large variability between patients in the actual time the measurement were taken can be taken into account. This is done by including the continuous time variable in the model.

A particular study design is motivated by the aims and the settings of the intervention. An intervention limited in time because performed in an inpatient medical institution (hospital or rehabilitation clinic) may have good short-term effects but the long-term effects are to be evaluated. For long-term community based interventions, the dynamic of the intervention may be more of interest. We illustrate the limitations of various approaches encountered in the review with the physical functioning score at three weeks, six, 12, and 15 months of the FS-36 questionnaire from the BOMeN study.

### Reduction to mean

We illustrate the effects reduction to the mean using a mixed model to estimate the overall effect of the intervention over time. Reduction to the mean occurs when there are some extreme outcome values at baseline which will see stronger effects than the values close to the mean. Consider three subsets of our dataset: A. only patient with scores in a middle range; B. patient with score in a middle to upper range (add patients with worse conditions at baseline than in A); C. score in a lower and middle range (add patient with better conditions at baseline than in A). An overall reduction in score (negative regression coefficient) indicates an overall better physical functioning.

We then compare the group effects: 1. baseline outcome values are not a covariate and 2. baseline outcome values are a covariate in the model. For data A, the difference in effect between 1. (−0.107 (0.064)) and 2. (−0.112 (0.067)) is small because the baseline values are homogeneous. For data B (1. -0.036 (0.049) and 2. - 0.028 (0.054)) and data C (1. (−0.019 (0.066) and 2.−0.047 (0.059)) the differences are respectively almost a third less and twice more when baseline values are adjusted for (Table 4). This is because in data C, the patients with perfect physical functioning at baseline saw no improvement even if the intervention did have an effect on those who could improve. Not adjusting for baseline meant underestimating the true effect. In data B. the difference is due to the stronger effect seen on patient with the higher scores. Not adjusting for baseline meant overestimating the true effect. In data A, the effects of the reduction to the mean are limited because no patient had extreme scores. The adjusted effects are different in the three datasets because they are obtained on different populations.

**Table 4:** Illustration of the effect reduction to the mean when no adjustment for baseline outcome values is performed

Patient characterisation at baseline		Intervention’s effect (standard error) obtained with:		Consequence of the reduction to the mean
	N	No adjustment for baseline values	Baseline values as a covariate	
Middle condition	141	−0.107 (0.064)	−0.112 (0.067)	No major consequence
Middle + worse condition	173	−0.036 (0.049)	−0.028 (0.054)	Effect overestimated
Middle + better condition	181	−0.019 (0.066)	−0.047 (0.059)	Effect underestimated

### Difference from baseline

Redefining the outcome as difference from baseline is problematic and can be easily avoided. If the data is analysed longitudinally, then the outcome value has a different meaning at each time-point due to the varying time laps between baseline and time-point. If the analysis is cross-sectional (i.e., one post intervention measure) then the best approach is to have baseline as a covariate in the model [2].

A slope model is suitable to measure the score change over time. This model provides an overall rate of change per time unit from baseline or from the first post intervention measurement. It is measured by the slope of the fitted line relating outcomes values to the continuous time. It will show if in the intervention group the physical functioning scores increase faster than in the control group. The difference in slope between the groups is obtained by estimating a parameter for time-group interaction. The rate of change per time unit is estimated from baseline, then baseline is an outcome time-point. Using physical functioning score at baseline, three weeks, six, 12, and 15 months of the FS-36 questionnaire data from the BOMeN study, we obtained (Table 5) that the mean score decreases by 0.0011 score points per week more in the intervention group than in the control group (confidence interval [−0.0004, 0.0026]).

**Table 5:** Results of data analysis for specific endpoints (N = 295)

Difference from baseline		
Group*time (continuous)^a^	−0.0011 (0.0006)	Difference between the groups in score decrease per week.
Cross-sectional effect at each time point		
Group difference at t1	Change in group difference t2 - t1	Change in group difference t3 - t1
Group	Group*t2 (categorical)^a^	Group*t3 (categorical)^a^
−0.098 (0.075)	0.003 (0.081)	0.031 (0.085)

^*^Interaction term

### Cross-sectional analysis at each time-point

Cross-sectional analysis at each time-point should be avoided because the dynamic within each patient is lost. There is also a loss of power due the necessary correction for multiple testing. A correct procedure is to use a mixed model with time as categorical variable with time-group interactions and baseline outcome values as a covariate. The model provides an estimate of the intervention effect at each time-point making a maximal use of the data available (Table 5).

Using data from the BOMeN study we obtained that the intervention group had a score lower by 0.097 (SD: 0.072) score points than the control group at the first time-point (three week). Then at six month this difference was decreased by 0.0002, i.e., unchanged compared to three weeks. Then at twelve months, the difference between the groups is decreased by 0.033 score points compared to the first time-point to −0.064 score points. This means that the maximum effect of the intervention is seen directly at the end of the intervention (three weeks) and is sustained the first six months and then decreases.

## Conclusion and suggestions

Our review has shown that not only the reporting of RCTs in the rehabilitation post-stroke needs improvement (see recommendation of the CONSORT statement [13]) but also the method of analysis itself. A lot of collected information was lost. More methods are available for analysing longitudinal data which were not discussed here [6, 11]. We have attempted, using real data as an example, to show the consequences of using some of approaches which are sub-optimal. We also showed how results of a regression analysis can be presented in an informative way using regression parameters and confidence intervals. We recommend that, despite limited publication space, the primary research question should be clearly stated and the overall intervention effect over the duration of follow-up should always be reported. Secondly the intervention effect at the particular follow-up measurements (estimated from a longitudinal model with time represented by dummy variables and the interaction between time and the intervention variable) can be reported. Also by using time as a continuous variable an estimate of the overall rate of change can be obtained. This also applies to study protocols. All covariates and the method of adjustment for baseline should also be clearly indicated because they influence the estimated intervention effect.

## Additional files

Additional file 1:
**Search Terms.**
Additional file 2:
**Flowshart.**
