# Supplementary material for: Longitudinal randomised controlled trials in rehabilitation post-stroke: a systematic review on the quality of reporting and use of baseline outcome values
Source: BMC Neurol. 2015 Jul 1;15:99. doi: 10.1186/s12883-015-0344-y (PMC4488053; doi:10.1186/s12883-015-0344-y)
Supplement: Additional file 1: — Search Terms. [file 12883_2015_344_MOESM1_ESM.pdf]

MEDLINE

RCT	Review	Functioning	Bereich	Reha
Randomized Controlled Trial" [Publication Type]	NOT "Review" [Publication Type]	Funct* OR functioning	„Stroke” OR "Stroke"[Mesh] OR „Brain Injurie?“ OR „Brain Injuries“[Mesh]	„Rehabilitation” OR "Rehabilitation"[Mesh] OR Rehab*

human AND "Randomized Controlled Trial" [Publication Type] NOT "Review" [Publication Type] AND (Funct* OR functioning) AND ("Stroke" OR "Stroke"[Mesh] OR "Brain Injuries" OR "Brain Injuries“[Mesh]) AND ("Rehabilitation" OR "Rehabilitation"[Mesh] OR Rehab*)

-->1136 Ergebnisse

Medpilot

(RCT OR "Randomized Controlled Trial" OR "Randomisierte kontrollierte Studie" OR "Clinical Trial" OR "Klinische Studie") AND (function* OR functional OR functioning) AND (Stroke OR "Cerebrovascular accident" OR Schlaganfall OR Apoplex OR "Brain Injuries" OR "Brain Injury" OR "intracranial injury" OR "Schädel-Hirn-Trauma" OR Gehirnverletzung) AND (rehabilitation OR rehab*) AND DT=ARTIKEL

-->578 Ergebnisse

Medline 564
14 nicht MEDLINE

Cochrane Library

ID	Search	Hits
#1	"randomized controlled trial":pt (Word variations have been searched)	325572
#2	functional or functioning:ti,ab,kw (Word variations have been searched)	79838
#3	MeSH descriptor: [Stroke] explode all trees	4516
#4	stroke:ti,ab,kw (Word variations have been searched)	19119
#5	MeSH descriptor: [Brain Injuries] explode all trees	949
#6	"brain injury":ti,ab,kw (Word variations have been searched)	1575
#7	MeSH descriptor: [Rehabilitation] explode all trees	13023
#8	rehab or rehabilitation:ti,ab,kw (Word variations have been searched)	9541
#9	#1 and #2 and (#3 or #4 or #5 or #6) and (#7 or #8)	781

25 nicht MEDLINE

Scopus

ScienceDirect

TITLE-ABSTR-KEY((randomi?ed controlled trial)) and TITLE-ABSTR-KEY(rehabilitation) and

(TITLE-ABSTR-KEY(**stroke**) or TITLE-ABSTR-KEY(**brain injury)**)
and
(TITLE-ABSTR-KEY(**functional**) or TITLE-ABSTR-KEY(**functioning**))

75 articles found for: TITLE-ABSTR-KEY(**(randomi?ed controlled trial)**) and TITLE-ABSTR-KEY(**rehabilitation**) and (**TITLE-ABSTR-KEY(stroke) or TITLE-ABSTR-KEY(brain injury)**) and (**TITLE-ABSTR-KEY(functional) or TITLE-ABSTR-KEY(functioning)**)[All Sources(Medicine and Dentistry,Nursing and Health Professions,Pharmacology, Toxicology and Pharmaceutical Science)]
