# Supplementary material for: Longitudinal randomised controlled trials in rehabilitation post-stroke: a systematic review on the quality of reporting and use of baseline outcome values
Source: BMC Neurol. 2015 Jul 1;15:99. doi: 10.1186/s12883-015-0344-y (PMC4488053; doi:10.1186/s12883-015-0344-y)
Supplement: Additional file 2: — Flowshart. [file 12883_2015_344_MOESM2_ESM.pdf]

PRISMA 2009 Flow Diagram

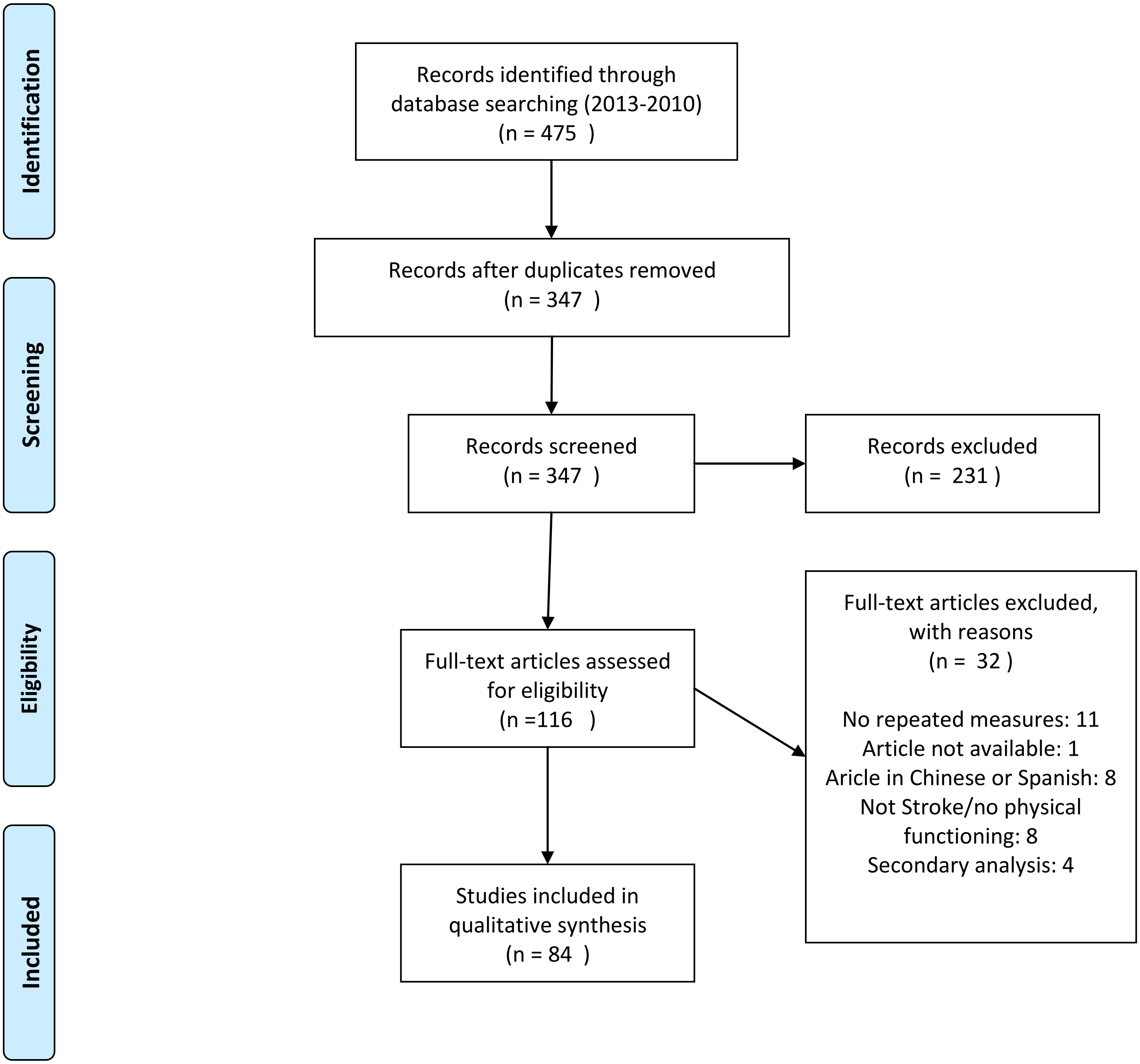

From: Moher D, Liberati A, Tetzlaff J, Altman DG, The PRISMA Group (2009). *P*referred *R*eporting *I*tems for *S*ystematic Reviews and *M*eta-*A*nalyses: The PRISMA Statement. PLoS Med 6(6): e1000097. doi:10.1371/journal.pmed1000097

For more information, visit www.prisma-statement.org.
